# Human Electroretinal Responses to Grating Patterns and Defocus Changes by Global Flash Multifocal Electroretinogram

**DOI:** 10.1371/journal.pone.0123480

**Published:** 2015-04-13

**Authors:** Man Pan Chin, Patrick H. W. Chu, Allen M. Y. Cheong, Henry H. L. Chan

**Affiliations:** Laboratory of Experimental Optometry (Neuroscience), School of Optometry, Hong Kong Polytechnic University, Hong Kong SAR, China; University of Florida, UNITED STATES

## Abstract

The electrical response of the retina was examined as a function of retinal region, using stimuli of various spatial frequencies in the first experiment. In the second experiment, the regional response of the retina to defocus at high and low spatial frequencies was investigated. Twenty three subjects were recruited for global flash multifocal electroretinogram (mfERG) in experiment 1. Black and white gratings (printed on plastic transparent sheets) of four spatial frequencies (SF), 0.24, 1.2, 2.4 and 4.8 cycle per degree were presented in front of the mfERG stimulation. The amplitudes and implicit times of the direct (DC) and induced (IC) components of mfERG responses were pooled into six concentric rings for analysis. There was low amplitude DC at low SF, which increased with increasing SF, and which decreased with increasing eccentricity. The IC was high in amplitude at all SF and reduced in amplitude with increasing eccentricity. Our findings suggested that outer and inner retina had different characteristics in processing spatial details. In experiment 2, Twenty-three young adults were recruited for mfERG measurement. The retinal electrical responses for low (0.24cpd) and high (4.8cpd) SF under fully corrected conditions of short-term negative defocus (-2D) and short term positive defocus (+2D) conditions were measured. There was a sign-dependent response to defocus in the DC response, mainly in peripheral regions. The sign dependent response at low SF was more obvious than that at high SF, and was located more peripherally. The IC response showed no clear trends for either defocus condition. The human retina seems to have a decoding system for optical defocus, which was tuned for low spatial frequency, and was located in the retinal near periphery.

## Introduction

With the exceedingly fast growing population of myopia (short-sightedness) in the world, myopia becomes an important global public health problem [1–3]. In the past few decades, many studies have improved our understanding on myopia development, in particular the factors affecting the eye growth and refractive errors. The retina, as the first site of the visual pathway to receive visual signal, has been shown to be the locally controlled of eye growth [4–6], which is tightly influenced by visual experience. Partial/full deprivation of vision results in unconstrained elongation of the eyeball, which corresponds to the myopia development, in animals (e.g. chicks[7, 8], fish[9], guinea pig[10], mice[11], marmoset[12], rhesus macaque[13], tree shrew[14, 15]) and human[16, 17], reflecting the importance of visual stimulus for the regulating eye growth. However, what types of visual stimulus are required for regulating the emmetropization (i.e. free from refractive errors)? Visual stimulus projected onto the retina comprises a wide range of spatial frequencies, which plausibly tune the emmetropization [18–22]: Schmid and Wildsoet [21] found that exposure to spatial frequency within the range of 0.086 and 4.3 cycle per degree could inhibit form-deprived myopia in chick [21]. Although spatial frequency dependency of emmetropization appears to be tuned to middle spatial frequencies in animals [18, 19, 21, 22], it is unclear whether similar spatial-frequency dependence occurs in human. Imposing defocuses or form deprivation at a local retinal area promotes regional eye growth [4–6], suggesting the predominance of local compensatory activity with feedback mechanism in regulating the eye growth. Compelling evidences have demonstrated the significant role of retina in eye growth regulation (refer to Wallman [23] for a review). First, although the eye growth, as shown in the animal models, can be regulated by the imposed defocuses [24, 25], it remains unclear how the retina decodes the sign and magnitude of defocuses. It is hypothesized that the retinal processing of eye growth-modulating visual stimuli relates to the activity of the amacrine cells [26, 27]. Second, several biochemical messengers regulating the eye growth have been identified within the retinal level. The relationship between sign-dependent changes in the messengers’ concentration and different signs of defocus (dopamine [28, 29], retinoic acid [30, 31] and glucagon [32, 33]) further supports that the retina responds to the signs of defocus. This compensatory activity manipulates the eye growth to achieve the clear retinal image. It is a kind of local self-regulation for the adaptation to the environmental influence. More understanding on it will help to find out how self-regulation works in the retina. Since there is limited study for human, studying the retinal physiological interaction between the visual stimulus and optical defocus in human eye allows better understanding of the underlying mechanism in eye growth.

The multifocal electroretinogram (mfERG) measures electrical activity of multiple retinal loci in response to light stimuli [34] and would be helpful in investigating how retina response to visual stimulus and defocus in various retinal regions. Ho and co-workers [35] measured the global flash (MOFO) multifocal electroretinogram (mfERG) in humans and noticed different physiological characteristics were obtained under hyperopic and myopic defocus conditions. In addition, peripheral retina showed a more vigorous change than central retina in response to optical defocus. In this study, we applied this methodology to examine the changes in retinal activities responding to specific spatial frequencies under different defocus conditions.

We speculate that if emmetropization is spatial- and local-dependent, the human retina ought to respond differently between visual stimuli with various spatial frequencies. In experiment 1, retinal electrophysiological response in different retinal regions related to stimulation with gratings of different spatial frequency (SF) was investigated. Furthermore, the retinal activities related to specific spatial frequencies under various defocus conditions have not been studied. In experiment 2, the mfERG responses related to different defocus conditions with high and low SF stimuli were studied. While the bi-directional optical axial length changes were observed in response to defocus in young adults [36], we believe that studying the electrophysiological response of young adults can reflect the neural response to defocus prior to eye growth. In addition, the ocular status of young adult is more stable as compared to the children. According to Ho *et al*. [35], the children at the age with myopia progression would have physiological changes in retina, especially at the fovea, which would influence the investigation on the interaction between visual stimulus and optical defocus [37].

## Methods

### Experiment Setup

**Fig 1:**
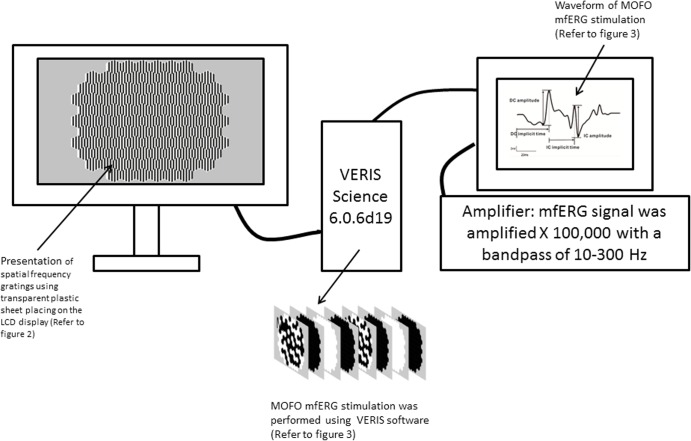
The schematic diagram of mfERG setup for experiment 1 and 2. The spatial frequency (SFs) gratings were presented on a 22-inch LCD display. The global flash mfERG stimulation was driven by VERIS Science 6.0.6d19. The mfERG signal was amplified X 100,000 with a bandpass of 10–300 Hz.

The schematic diagram of mfERG setup for experiment 1 and 2 is shown in Fig 1. The spatial frequency (SFs) gratings (refer to “Presentation of grating stimuli” session) were presented on a 22-inch colour liquid crystal display monitor (Model: VX2260wm, ViewSonic, Hong Kong, China) during the mfERG stimulation. The mfERG stimulation (Refer to “Global flash mfERG stimulation” session) was performed using the Visual Evoked Response Imaging System software (VERIS Science 6.0.6d19; Electro-Diagnostic Imaging Inc., San Mateo, CA, USA). The stimulus array consisted of 103 non-scaled hexagons and the stimulus pattern subtended 33.7° vertically and 38.4° horizontally at a viewing distance of 40 cm. Non-scaled hexagons were used in this study to maintain the same SFs at different eccentricities.

### Presentation of grating stimuli

**Fig 2:**
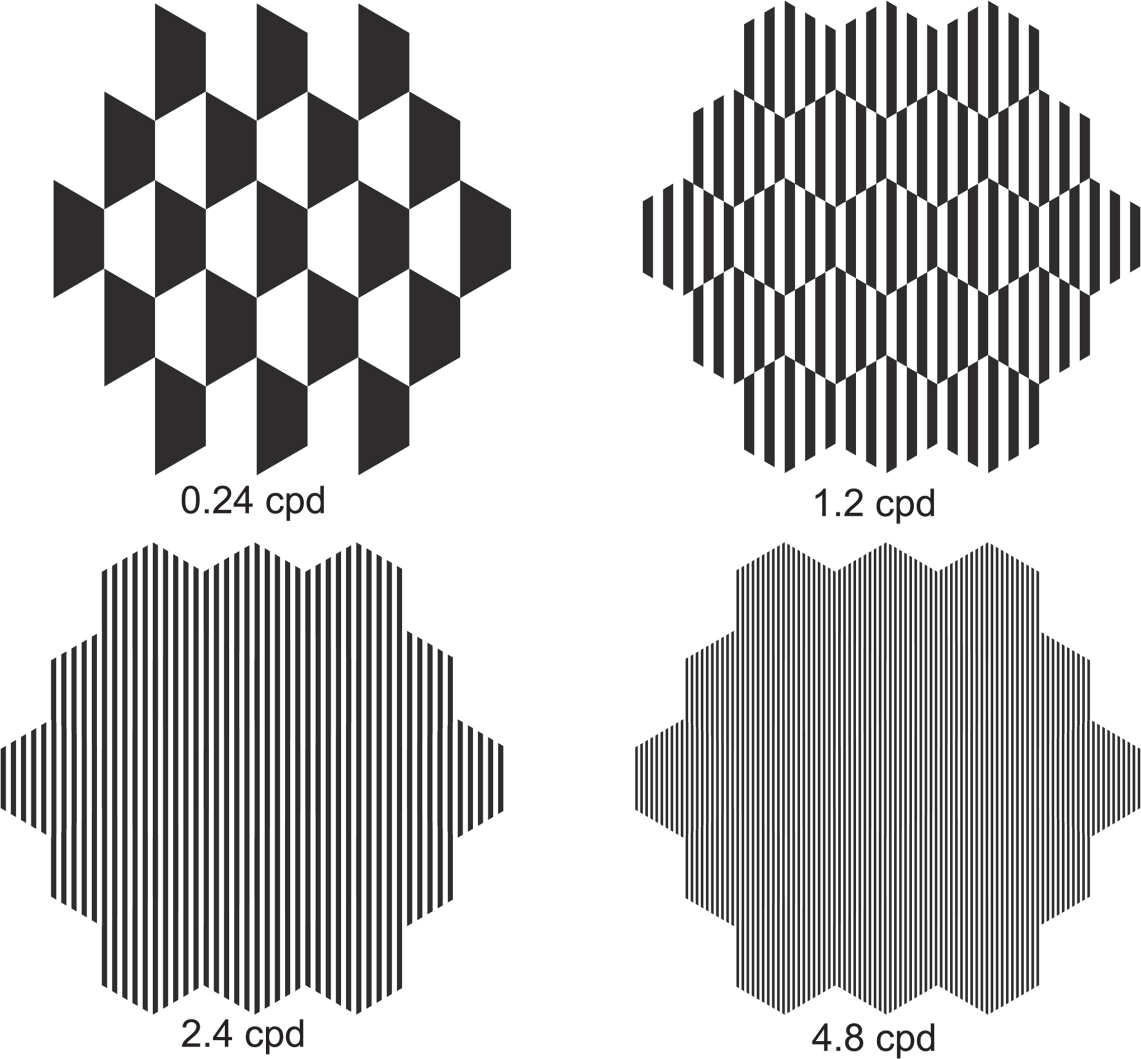
Spatial frequency stimuli presented to subjects 1 for mfERG. Spatial frequencies of 0.24, 1.2, 2.4 and 4.8 cycle per cpd was presented. Only ring 1 to 3 were shown in the figure.

The gratings were drawn precisely using Adobe Illustrator CS4. The outline of 103 hexagons was incorporated into the software. The gratings were precisely drawn and printed on transparent film as alternating black and transparent stripes within each hexagon. The widths of the gratings were 14.4, 2.88, 1.44 and 0.72 mm, corresponding to spatial frequencies of 0.24, 1.2, 2.4 and 4.8 cycle per degree (cpd) at 40 cm (Fig 2). The spatial frequencies selected in this experiment is below nyquist limit of photoreceptors and ganglions[38], so 4.8cpd can still be “resolved” by both outer and inner retina at peripheral 20. The luminance variations for different size of gratings in each hexagon during mfERG flash were minimized by presenting equal areas of black stripes in all gratings. The luminance of individual hexagon overlaying the gratings was measured using a spectro-radiometer (Model: SR3, Topcon, Japan). The luminance of whole individual hexagon was tried to measure by orientating size field of spectro-radiometer just within the individual hexagon. The luminance was found approximately the same whenever gratings were used.

### Global flash mfERG stimulation

**Fig 3:**
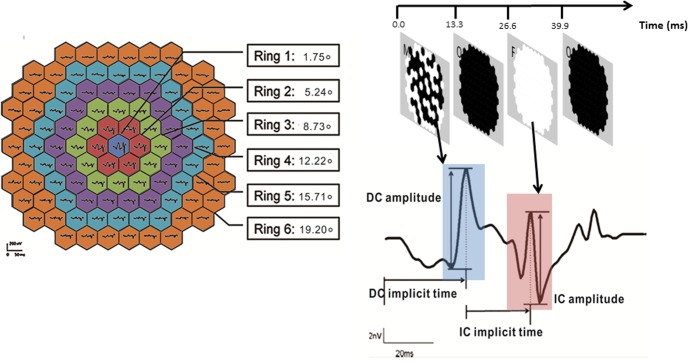
Schematic diagram for grouping the mfERG responses and the waveform of MOFO mfERG. Left: Both DC and IC responses were pooled into concentric rings with eccentricity from ring 1 (0°) to ring 6 (19.20°)Right: Schematic diagram for DC amplitude and implicit time, IC amplitude and implicit time of global flash mfERG.

High contrast (96% contrast; bright phase, 180 cd/m^2^; dark phase, 4 cd/m^2^) global flash (MOFO) mfERG was selected to measure the retinal activity. In each cycle of MOFO mfERG stimulation, consisted of a pseudo-random focal flash (M), followed by a full-screen dark frame (O), a full-screen global flash (F), and another full-screen dark frame (O) (Fig 3). This can enhance the activity from inner retinal neurons, in order to obtain separate outer and inner retinal responses [35, 39–44]. In this study, as grating patterns were presented on the display during the measurement of each MOFO cycle, gratings were seen during the bright frames, thus M frame and F frame, while it became dark in the dark frames. And half the area of each hexagon was covered by dark strips of the gratings, the luminance of was reduced to half during M frame and F frame. All the grating patterns had the same number of black and white strips within each mfERG hexagonal stimulus and hence the measured luminance of different grating patterns was the same. The ratio of global flash to focal flash was maintained at 1:1 in this study to obtain both optimal DC and IC responses [45]. The MOFO mfERG results in a direct component (DC) and an induced component (IC) which can be separately measured to assess the outer and inner retinal responses. In addition, regional variations of the responses can be observed in the same measurement period.

### Recordings

The mfERG responses were recorded using a Dawson–Trick–Litzkow (DTL) thread electrode, which was positioned on the inferior cornea along the lower lid margin. Gold-cup reference and ground surface electrodes were placed at the outer canthus of the tested eye and at the forehead respectively. The pupil of the tested eye was dilated (>7mm) using 0.5% Tropicamide (Alcon, Australia) and the tested eye was fully corrected for the viewing distance of 40cm during the mfERG recording. The mfERG signal was amplified X 100,000 with a bandpass of 10–300 Hz (Model: 15A54, Physiodata Amplifier system, Grass Technologies, Astro-Med, Inc., West Warwick, RI, USA). With the 2^12^ binary m-sequence used, the recording time for each condition was about 7 min and 17 sec. The recording was divided into 32 slightly overlapping segments, and subjects were permitted to rest between segments. Subjects were instructed to fixate the red-cross target in the middle of the central hexagon of the stimulus display. The electrical signals were monitored by the examiner using the real-time display provided by the VERIS system and any segments contaminated with blinks or other artifacts were discarded and re-recorded.

### Subject recruitment

The criteria for recruiting subjects were the same in both experiment 1 and 2. Young adults were recruited from The Hong Kong Polytechnic University. The range of refractive errors was from +1.50D to -4.00D and astigmatism was not more than -1.25D; it is known that high myopia may affect mfERG findings [40]. Detailed eye examination was conducted before the experiment. Intraocular pressure and depth of anterior chamber angles were assessed to ensure suitability for dilated fundus examination and mfERG measurement. Subjects with colour vision deficiency, retinal ocular diseases, abnormal ocular media, history of general diseases that have potential ocular effects, or a history of photosensitive epilepsy were excluded from participation. The tested eye was randomly chosen. Tested eyes had best corrected logMAR visual acuity of 0.00 (Snellen acuity of 6/6) or better. After detailed explanation of the study, written informed consent was obtained from each subject; the study and the consent form was reviewed and approved by the Human Ethics Committee of The Hong Kong Polytechnic University (HSEARS No. 20110905002) and adhered to the tenets of the Declaration of Helsinki. Subject information was anonymized and de-identified prior to analysis.

#### Experiment 1: Effect of grating stimulus on global flash mfERG at different retinal regions

Twenty three young adults aged from 21 to 27 years (mean = 22.5 ± 1.6 years) participated in the study. The refractive errors (spherical-equivalent) of the subjects ranged from +1.13 to -3.25D (mean = -1.02 ± 1.13D) and astigmatism ranged from 0.00D to -1.25D (mean = -0.44 ± 0.44D). The tested eye of the subjects was fully corrected for the 40 cm viewing distance using 35mm diameter trial lenses. The retinal image size among the subjects was kept essentially constant by placing the corrective lenses at the anterior focal plane of the tested eye.

#### Experiment 2: Global flash mfERG response to grating under defocus conditions at different retinal regions

Twenty-three young adults aged from 20 to 27 years (mean = 22 ± 1.7 years) were recruited. The refractive errors (spherical-equivalent) of the subjects ranged from +1.13D and -2.50D (mean = -0.48 ±0.89D) and astigmatism ranged from 0 to -1.00D (mean = -0.28 ± 0.34D). Two drops of 1% Tropicamide (Alcon Laboratories Inc., Fort Worth, TX, USA) were instilled with a 5-min interval to achieve the cycloplegic effect, and cycloplegia was evaluated as discussed below. Low and high SF gratings, 0.24cpd (low) and 4.8cpd (high), were selected, and the mfERG was then measured for each under various optical defocus conditions, including plano (fully corrected at 40cm), positive defocus (+2D) and negative defocus (-2D). When imposing -2D defocus, the residual accommodation was compensated. For example if a subject possessed 0.5D residual accommodation, a -2.5D lens was used to impose -2D defocus. All subjects were reminded to report if they could manage to fixate the red-cross fixation before and during recording. Since Wang and colleagues [46] showed the peripheral resolution was limited by neural sampling density, but not by several diopter of defocus, we supposed the gratings could be resolved by retina with +/- 2D of defocus at all regions.

#### Evaluating the cycloplegic effect and residual accommodation

In Experiment 2, the cycloplegic effect and the residual accommodation were assessed to ensure that they were constant throughout the experiment, as in the study of Ho *et al*. [35] The push-up method was employed to measure the residual accommodation of the tested eye of all subjects. In the residual accommodation measurement, the subjects’ refractive errors were corrected by adding +2D for near. A line of letters at their best acuity was gradually moved from a working distance of 50 cm toward the subject. The end point was reached when the line of letters was reported to blur. The residual accommodation was calculated by subtracting 2D (the near addition power given) from the amplitude of accommodation measured. This procedure was carried out three times 20 min after the instillation of the eye drops, and immediately before and after the mfERG measurements by a masked examiner. The mfERG recordings were continued only if the difference in residual accommodation for three consecutive measurements, each separated by about 1 minute, was not more than 0.25D.

## Data Analysis

First order kernel response of direct (DC) and induced (IC) components of mfERG responses were extracted and pooled into six concentric rings for analysis (Fig 3, left panel). For the central ring (1), the radius angle subtended 1.75°and the most peripheral ring (6), the radius angle subtended 19.20°. The amplitude of the DC was defined as the difference from the first negative trough to the first positive peak while the IC amplitude was defined as the difference from the second positive peak to the second negative trough [35, 41, 43, 47]. The implicit time of the DC was defined as the time to reach the first positive peak and the implicit time of the IC was measured from the presentation of the global flash to the second positive peak (Fig 3, right panel).

Predictive Analytics SoftWare (PASW19.0, SPSS Inc., Chicago, IL, USA) was used for data analysis. Repeated measures analysis of variance (ANOVA) was applied to study the effect of spatial frequencies on mfERG responses (Experiment 1) and the effect of spatial frequencies with optical defocus on mfERG response (Experiment 2) for different retinal regions. Post hoc tests with Bonferroni adjustment were applied to correct the level of significance due to multiple comparisons of different retinal regions. The level of significance was set at 0.05.

## Results

### Experiment 1. Effect of grating stimulus on global flash mfERG at different retinal regions

**Fig 4:**
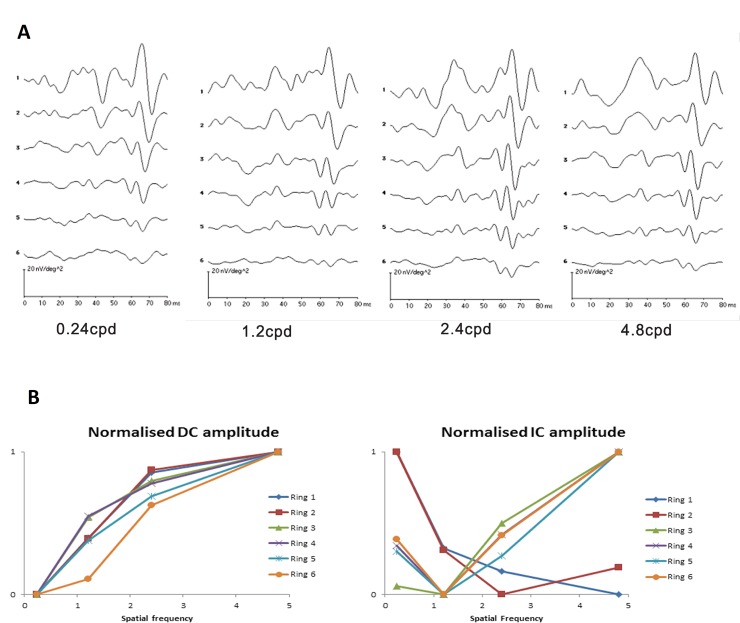
The global flash mfERG response to spatial frequency. (A) The typical global flash mfERG waveform measured from one subject with 4 spatial frequencies grating for six different retinal regions. (B) Normalized amplitudes of DC and IC response to spatial frequency of all subjects. The normalized amplitudes of DC and IC were achieved by dividing the amplitudes by the maximum value in each ring.

The typical waveforms of the global flash mfERG for the four grating patterns are shown in Fig 4A.

The first and second peaks of the waveforms are the DC and IC respectively.

There is low amplitude DC with low SF grating (0.24cpd), the DC amplitude increases with increasing number of gratings, and the DC amplitude decreases with increasing eccentricity at all eccentricities.

The IC is high in amplitude for all grating SFs and reduces in amplitude with increasing eccentricity. The IC waveform alters from a biphasic peak-trough formation in the central retina to a much-reduced triphasic form with reduced amplitude in the periphery. The retinal response to spatial frequency was illustrated by the summary normalizing DC and IC amplitude (Fig 4B). For DC, the responses to spatial frequency in central and peripheral regions were similar. While for IC, the central retinal response to spatial frequency was different from peripheral retinal response.

**Fig 5:**
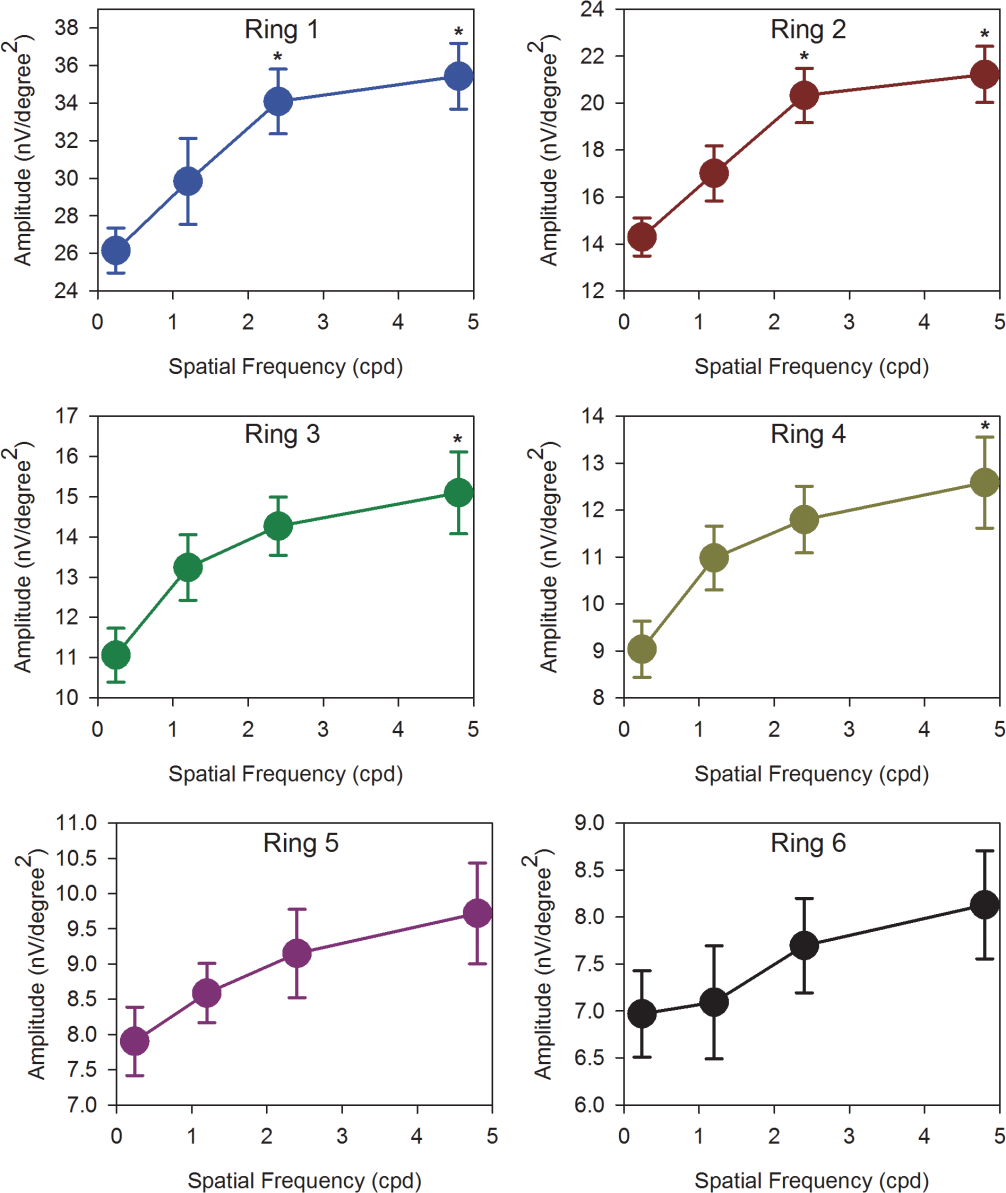
DC amplitudes. Absolute amplitudes of DC (mean ± SEM) with SF of 0.24cpd to 4.8cpd of ring 1 to 6. The DC responses were generally increased with SF in all regions. Those marked with an asterisk ‘‘*” are statistically different from the 0.24cpd.

**Fig 6:**
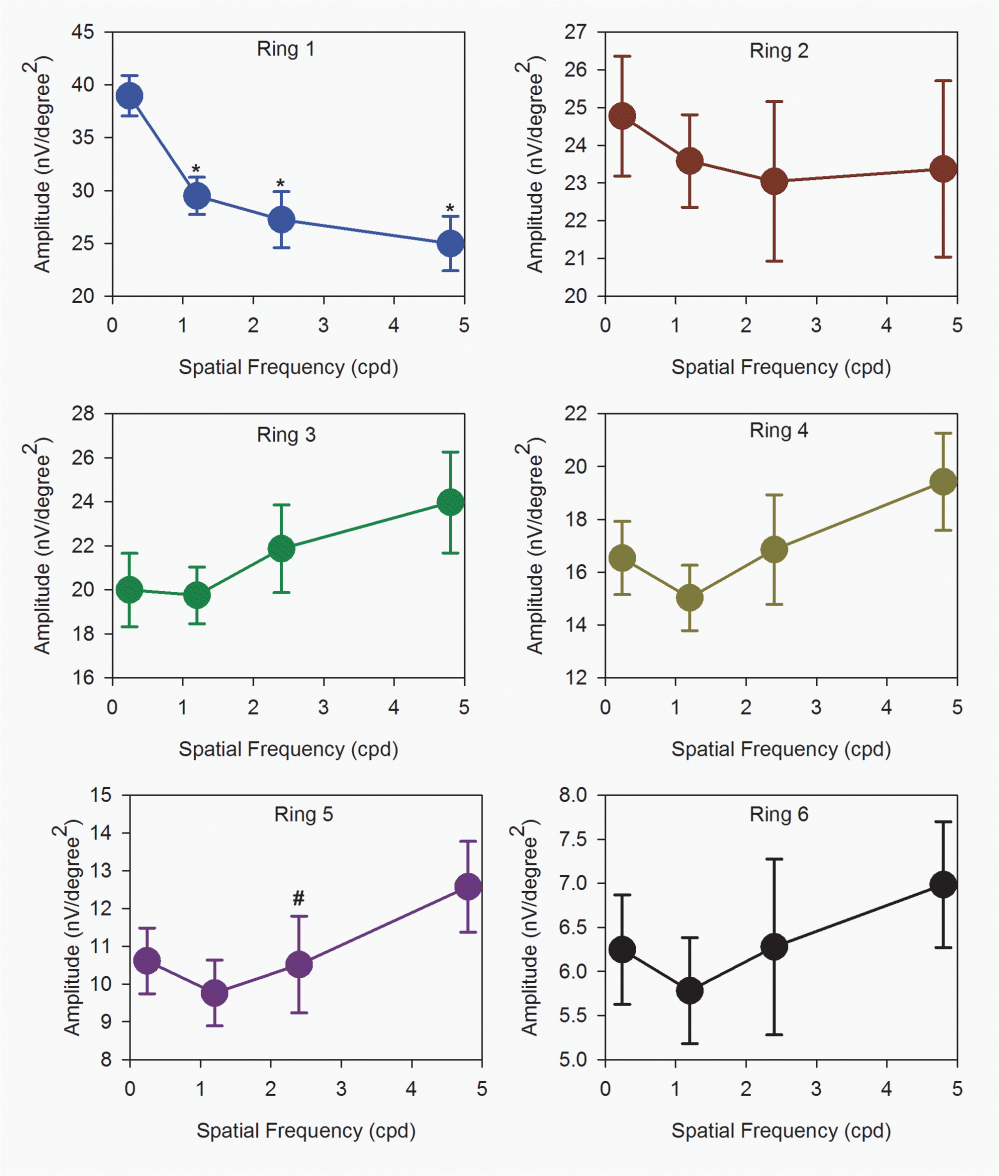
IC amplitudes. Absolute amplitudes of IC (mean ± SEM) with SF of 0.24cpd to 4.8cpd of ring 1 to 6. In ring 1 and 2, the IC responses were similar. While from ring 3 to 6, the IC responses behaved differently from the central retina. Those marked with an asterisk ‘‘*” are statistically different from the 0.24cpd while with an asterisk“#” are statistically different between 4.8cpd and 2.4cpd.

When measurements are made across the 23 subjects in the group, these initial impressions are confirmed (Figs 5 and 6). The DC amplitudes are significantly different with both SF of gratings (2-way repeated measures ANOVA: (F = 14.36, p < 0.001)) and eccentricity (F = 351.67, p < 0.001). Fig 5 shows the relationship between DC amplitude and stimulus spatial frequency. For the central and paracentral retina (rings 1 and 2), DC amplitude increases significantly as spatial frequency is increased (Repeated measures ANOVA: ring 1: (F = 10.70, p < 0.001); ring 2: (F = 13.66, p < 0.001), In addition, the responses to 2.4 and 4.8 cpd stimuli are comparable. In the peripheral retina (rings 5 and 6), the relationship between DC amplitude and stimulus spatial frequency is essentially linear. Rings 3 and 4 appear to have a characteristic which is ‘transitional’ between the central and peripheral responses. In contrast, the IC amplitudes show two different characteristics with increasing SF (Fig 6). For the central retina (ring 1), IC amplitude is high with thick grating and decreases with increasing number of gratings. (Repeated measures ANOVA: (F = 16.65, p < 0.001)). A similar trend is observed in the paracentral (ring 2) region. For rings 3–6, amplitudes are low at low SF, and increase as SF increases. There is a significant main effect of eccentricity (2-way repeated measures ANOVA: (F = 105.15, p < 0.001)) in these data, while SF alone is not a statistically significant factor (F = 1.75, p = 0.18). However there is a significant interaction between SF and eccentricity (F = 13.11, p <0.001), indicating significantly different response amplitude profiles at different eccentricities.

DC implicit time reduced with increasing eccentricity, and repeated measures two-way ANOVA showed a significant main effect of eccentricity (F = 4.01, p = 0.017), but post-hoc analysis revealed few consistent effects between measurement points.

IC implicit time reduced with increasing eccentricity, and increased with increasing SF. Two-way repeated measures ANOVA showed significant main effects for eccentricity (F = 119.56, p <0.001) and SF (F = 6.14, p = 0.001). For all SF, post hoc analysis revealed the implicit time was significantly increased from ring 1 to ring 4 (all p <0.001). For ring 3, the implicit time for 1.2cpd was significantly longer than that for 0.24cpd (p <0.001). For ring 4, the implicit times for 1.2cpd were significantly longer than those for 0.24cpd (adjusted p <0.008) and 2.4cpd (p <0.001).

### Experiment 2. Global flash mfERG response to grating under defocus conditions for different retinal regions

**Fig 7:**
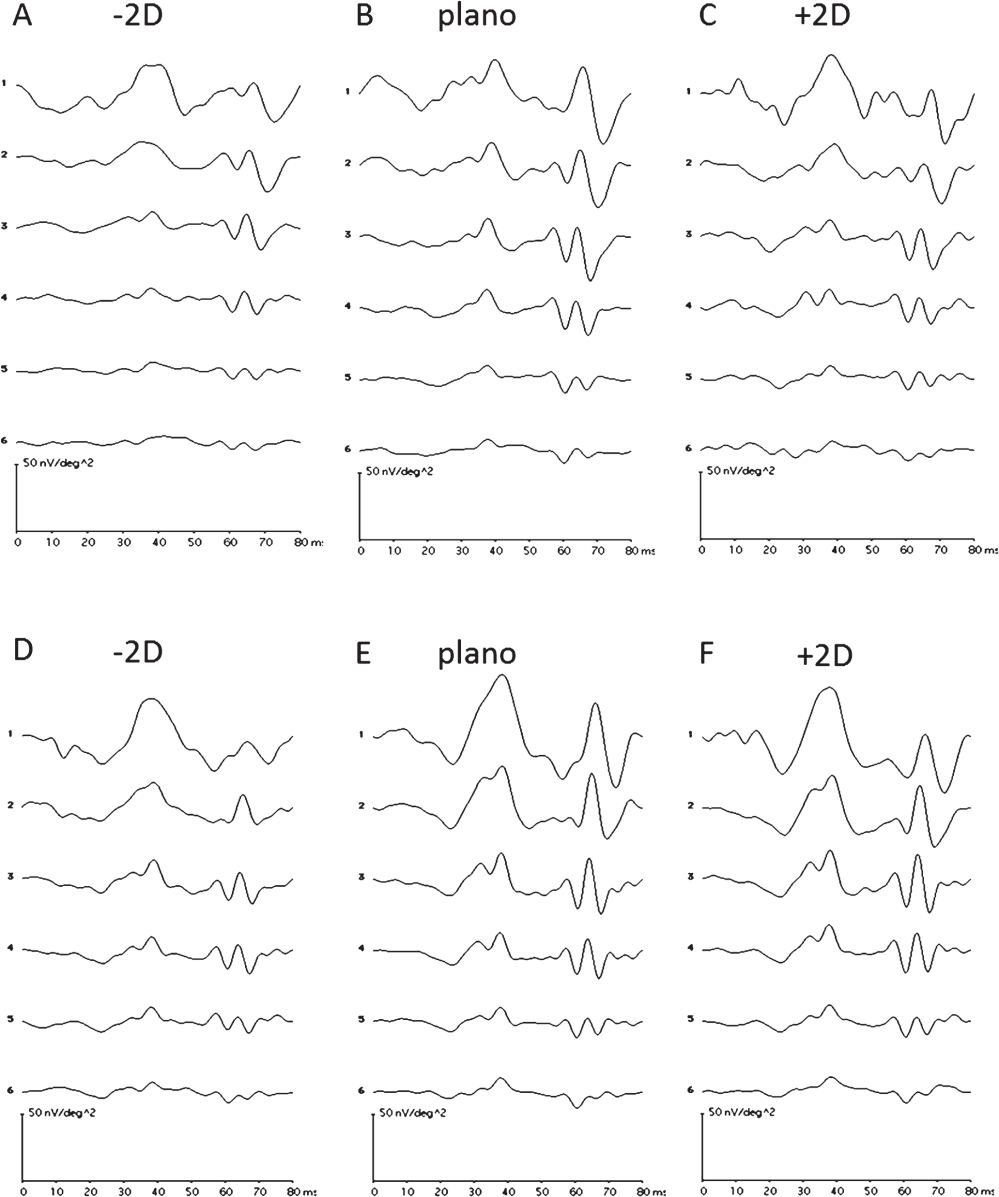
The typical global flash mfERG waveform from one subject for high and low spatial frequency under defocus. Upper panel: (A), (B) and (C) are responses from 0.24cpd under defocus of-2D, plano and +2D respectively. Lower panel: (D), (E) and (F) are response from 4.8cpd under under defocus of-2D, plano and +2D respectively.

The waveforms of MOFO mfERG DC and IC responses under defocus conditions for 0.24cpd and 4.8cpd are shown in Fig 7. At low SF (0.24cpd), defocus with -2D lenses broadens the DC response at all eccentricities, and reduces the amplitude of the response, especially in the retinal periphery. The IC response is diminished at all eccentricities (Fig 7A). Defocus with +2D lenses increases the DC response and decreases the IC response (Fig 7C). At high SF (4.8 cpd), defocus with -2D lenses decreases both DC and IC response, while defocus with +2D lenses appears to have much smaller effects on either DC or IC responses at any eccentricity (Fig 7 lower panels).

Table 1 shows the average DC and IC amplitudes (mean ± SEM) of ring regions when 0.24cpd and 4.8cpd stimuli are presented under different defocus conditions. The amplitude of both DC and IC decreases with increasing retinal eccentricity (from ring 1 to 6).

**Table 1:** Summary of amplitude (mean ± SEM) of DC and IC under different defocus conditions with 0.24cpd or 4.8cpd from ring 1 to 6.

	**DC**					
	**0.24cpd**			**4.8cpd**		
	**-2D**	**Plano**	**+2D**	**-2D**	**plano**	**+2D**
**Amplitude (nV/degree2)**						
**Ring 1**	31.26 ± 2.13	27.21 ± 2.14	32.36 ± 1.77	32.76 ± 2.27	34.32 ± 2.27	35.90 ± 2.35
**Ring 2**	17.04 ± 1.22	14.87 ± 1.14	17.91 ± 1.10	18.77 ± 1.31	20.44 ± 1.53	22.17 ± 1.65
**Ring 3**	11.32 ± 0.70	11.47 ± 0.67	13.79 ± 0.67	13.84 ± 1.12	14.29 ± 1.13	17.35 ± 1.19
**Ring 4**	9.58 ± 0.71	10.49 ± 0.64	11.32 ± 0.60	11.73 ± 0.85	11.49 ± 0.93	13.41 ± 0.89
**Ring 5**	7.66 ± 0.44	8.91 ± 0.60	9.53 ± 0.53	9.93 ± 0.55	9.83 ± 0.66	10.61 ± 0.62
**Ring 6**	6.59 ± 0.47	7.46 ± 0.51	8.10 ± 0.49	8.31 ± 0.43	8.72 ± 0.62	8.92 ± 0.64
	**IC**					
	**0.24cpd**			**4.8cpd**		
	**-2D**	**Plano**	**+2D**	**-2D**	**plano**	**+2D**
**Amplitude (nV/degree2)**						
**Ring 1**	28.95 ± 2.16	33.63 ± 2.54	31.65 ± 2.43	18.25 ± 1.78	22.29 ± 2.72	20.18 ± 2.91
**Ring 2**	21.72 ± 1.68	23.84 ± 1.77	24.97 ± 1.68	17.06 ± 1.52	21.62 ± 2.37	23.66 ± 2.54
**Ring 3**	19.05 ± 1.28	21.67 ± 1.87	23.02 ± 1.66	19.28 ± 1.77	20.83 ± 2.32	25.34 ± 2.62
**Ring 4**	15.23 ± 1.08	17.16 ± 1.44	17.56 ± 1.23	16.95 ± 1.67	17.30 ± 1.86	19.76 ± 2.04
**Ring 5**	9.67 ± 0.71	11.08 ± 0.88	10.64 ± 0.81	10.76 ± 1.01	10.99 ± 1.17	11.97 ± 1.13
**Ring 6**	6.44 ± 0.45	7.71 ± 0.61	6.11 ± 0.67	6.14 ± 0.64	6.24 ± 0.80	6.79 ± 0.69

**Fig 8:**
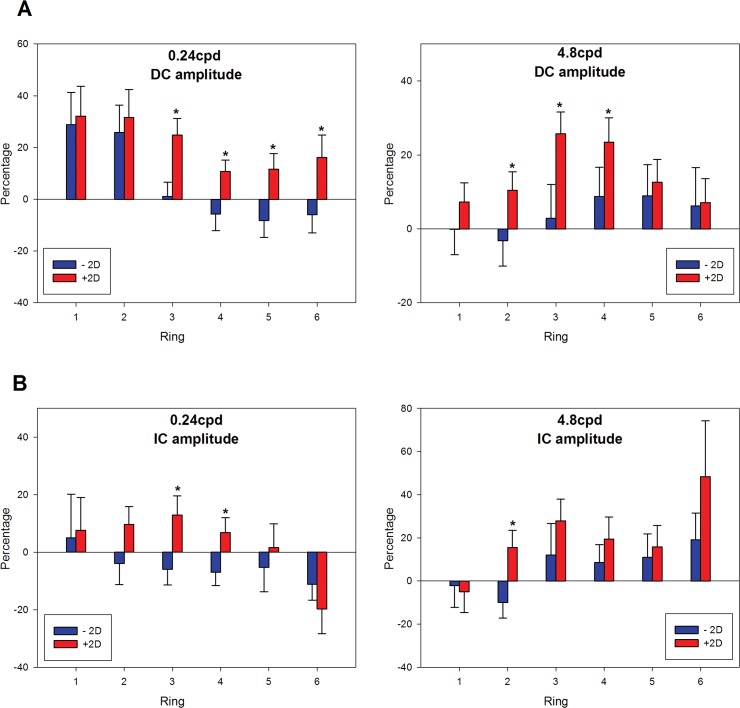
Percentage change of DC and IC with imposing defocus under 0.24cpd or 4.8cpd. (A) Percentage change of DC amplitudes (mean ± SEM) with imposing defocus under 0.24cpd or 4.8cpd across the region. (B) Percentage change of IC amplitudes (mean ± SEM) with imposing defocus under 0.24cpd or 4.8cpd across the region. Left panel is 0.24cpd and right panel is 4.8cd. Those marked with an asterisk ‘‘*” are statistically different between +2D and -2D defocus.

Changes for defocus with SF interaction are more complex, and are best seen as differences from the ‘in focus’ condition. The individual percentage changes of DC and IC, with positive or negative optical defocus comparing to plano condition for high and low SF, have been calculated and are shown in Fig 8A and 8B respectively. Although the eyes had mixed responses to defocus, the majority follows the pattern in Fig 8.

For DC (Fig 8A), the trend of amplitude change with +2D defocus is significantly different from that for -2D defocus (3-way repeated measures ANOVA: (F = 17.99, p < 0.001)), while SF and region alone had no simple effect (SF: F = 0.06, p = 0.80; Region: F = 1.63, p = 0.19). There was no significant 3-way (Region x SF x Defocus) interaction for DC amplitude percentage change (F = 1.98, p = 0.13). There was a significant interaction between region and SF (F = 3.94, p = 0.01), indicating differing response as eccentricity increases, as expected, but no interaction was found between region and defocus (F = 2.41, p = 0.07). Further examination of the region and SF interaction by 2-way repeated measure ANOVA revealed a significant region and SF interaction when imposing -2D defocus(F = 4.83, p = 0.005).

Imposing +2/-2D theoretically would produce the same magnitude of blur, and the retinal response would be the same if the retina responds only to blur and not to the sign of defocus. The result in this study demonstrates that imposing same magnitude of defocus with different sign produces different retinal responses. At low SF (0.24cpd), +2D defocus produces 20–30% increase in response at all eccentricities (Fig 8A, left panel). In contrast to this, -2D defocus gives increased amplitudes only centrally, but gives reduced amplitudes from the mid-periphery to the far periphery. Under 0.24cpd (Fig 8A left panel), both +/-2D can trigger significant increase in DC amplitudes at ring 1 and 2 compared to the plano condition (one sample T test, +2D: p = 0.01; -2D: p = 0.03) but the sign of defocus is not differentiable. From ring 3 to 6, +/-2D trigger increase/decrease according to sign of defocus. This characteristic is reduced at higher SF (4.8cpd). At 4.8cpd, the +2D defocus produces an increased DC amplitude in the near and mid-periphery, while the -2D defocus has essentially no effect on amplitude of response (Fig 8B, right panel).

The IC response appears to be more variable, but the +2D defocus at low SF shows increased response over the majority of the retina (excluding the far periphery), with strongest response in the mid-periphery. The -2D defocus shows a consistent decrease in amplitude of response across the peripheral retina (Fig 8B, left panel). Three-way repeated measures ANOVA of IC percentage change showed significant main effects of defocus (F = 7.60, p = 0.01), while region (F = 0.51, p = 0.65) and SF (F = 2.80, p = 0.11) did not show a main effect. There was a significant interaction between region and SF (F = 4.07, p = 0.01). There was no significant 3-way interaction (Region x SF x Defocus: F = 1.45, p = 0.24). At higher SF, the +2D defocus produced a consistent increase in IC amplitude across the peripheral retina, and smaller increases were seen with -2D defocus from ring 3 and beyond (Fig 8B, right panel).

For the DC implicit time, both SF and region had significant main effects (SF: F = 5.55, p = 0.003; Region: F = 6.29, p = 0.003) and there was no significant 3-way interaction (Region x SF x Defocus: F = 1.56, p = 0.19). For IC implicit time, both defocus and region had significant main effects (Defocus: F = 4.66, p = 0.03; Region: F = 72.28, <0.0001) but there was no significant 3-way interaction (F = 0.74, p = 0.48).

## Discussion

### Spatial frequency effect across the retina

In experiment 1, our findings demonstrated that the components of global flash mfERG responded differently from low to high spatial frequency. In addition, the response amplitude of DC and IC were not linear with width of the gratings. The DC amplitude increased rapidly from 0.24cpd to 2.4cpd and then slowed from 2.4cpd to 4.8cpd across the retinal eccentricity (Fig 5). However, the trend of IC amplitude was different. The IC amplitude was the highest at 0.24cpd and decreased with increasing SF at central (i.e. ring 1 and 2) (Fig 6). At the mid peripheral retina (from ring 3 to 6), the trends were opposite and the IC amplitude increased with SF. In the global flash mfERG, the DC response is the average response to a focal flash generated by m-sequences of the multifocal stimulus [41, 43, 47]. The amplitude mainly represents the activity from outer retinal composed of contributions from photoreceptors, and ON- and OFF-bipolar cells [39, 41, 43, 47, 48]. The global flash after the focal flash in the m-sequence stimulation produces the IC response, which represents the retinal adaptive response, and reflects activity from inner retina including amacrine cells and retinal ganglion cells [43, 44, 47, 49]. In this study, the luminance of the focal flash and the global flash was approximately constant while varying the SF and the ratio of global flash to focal flash was not changed. And the mean luminance of the stimuli at the different spatial frequencies was similar, the changes in DC and IC with SFs are not due to the difference of luminance of different SFs. The grating acuity is closely correlated to the receptive field size of RGC which is increased with eccentricity. However, the trends of DC and IC amplitude response to spatial frequency are not totally followed the variation of the RGC field size. Hence, the changes of mfERG responses may be only explained by the receptive field size partly. The different trends of DC and IC with increasing SF indicate that outer and inner retina may decode and process SF in different ways. The mfERG response pattern for central (rings 1 and 2) are very different to peripheral regions. We believe that the variations of the waveform with eccentricity may reflect different adaptive mechanisms across the retina. This regional change in responsiveness may be caused by variation in the rod/cone mix with eccentricity, change in the ways in which receptors and receptive fields are connected [41, 50].

Previous studies have used various ERG techniques to investigate the SF effect on retinal response [51–54]. Yamada and colleagues [54] applied focal macular ERG and found that the oscillatory potentials, which reflect the inner retina activity, decreased with increasing SF, and our findings are consistent with theirs. It has long been suggested that the retina has different channels which are selectively sensitive (tuned) to particular ranges of SF [55–58]. Our mfERG findings may support the idea of both outer [59–61] and inner [62–64] retina possessing spatial sensitivity to SF. In addition, our findings suggested outer and inner retina respond differently to SF in the central region, while this characteristic is reduced in peripheral regions.

Since refractive errors may affect the mfERG response, we recruited 12 age-matched subjects with spherical equivalent of -6.54±1.63D, going through the same procedure with experiment 1. The general trend of DC and IC amplitudes against spatial frequency for the high myope group were similar to those from low myope group (S1 Fig).

### Regional sensitivity to defocus

In experiment 2, the DC responses to +2D and -2D differ mainly in peripheral regions. For the 0.24cpd stimulus, the DC responses for rings 1 and 2 were less differentiable during stimulation with positive or negative defocus. In peripheral regions (ring 3 to 6), the DC responses were different with positive or negative defocus, suggesting that the peripheral region may be the site for the decoding of optical defocus. A similar hypothesis has been suggested in animal studies, that the retina may be able to decode defocus and the eye grows according to the sign of defocus [65, 66]. Subsequent studies have shown that peripheral defocus can influence overall refractive error development in chicks [67] or in monkeys [4, 68], highlighting the dominant role of peripheral retina in the regulation of ocular growth. In addition, myopic progression has been slowed by wearing orthokeratology lenses in clinical studies, and it has been speculated that the presence of myopic defocus on the peripheral retina may be the critical factor in slowing myopia development [69, 70]. In this study, IC percentage change in differentiating optical defocus in the peripheral retina can also be noticed in the 0.24cpd condition, but is not observed in the 4.8 cpd condition. This shows that sign-dependent responses to defocus are mainly from peripheral regions instead of central retina. Ho *et al*. [35] used MOFO mfERG in human eyes and showed that DC and IC demonstrated different trends of changes with various levels of defocus from -4D to +4D. The peripheral retinal response to defocus was more vigorous than that in the central retina, a finding in agreement with the present study. Furthermore, the subjects in this cohort and those in the study of Ho *et al*. [35] are mainly low stable myopes; the sign-dependent retinal characteristic is still present when myopia is stable. It has previously been noted that inner retinal function is different between stable myopes and progressing myopes [71]. It would be of interest to determine whether progressing myopes have similar regional retinal response characteristics to defocus and SF as those we have reported here.

In our findings, the +2D defocus stimulus generally triggered higher mfERG response than -2D defocus, suggesting that the retina responds more vigorously to myopic defocus. Previous animal studies have demonstrated that eye growth is more responsive to myopic defocus than to hyperopic defocus [72–74]. In addition, Zhong *et al*. [26] have shown that different conditions of optical focus can affect the activity of ON-bipolar as well as GABAergic amacrine cells in primate retina. They used immunocytochemical markers to illustrate cellular activity which increased when images were in focus or had positive defocus; the response decreased for images with negative defocus. These findings match our findings, that the mfERG responses, in both DC and IC amplitudes, are higher with +2D defocus than with -2D defocus over the regions under stimulation.

### Spatial frequencies and Defocus

Spatial frequency is a significant factor in our findings, even with the same amount of defocus in the same region. Our findings showed sign dependent characteristic was observed in the peripheral regions for both SF, and the DC reacted more vigorously under 0.24cpd (low SF) at ring 1 and 2 than those under 4.8cpd (high SF). Note that the low and high SF in this study is relative to each other, rather than resolution threshold of the retina. The vertical grating resolution limit of retina is about 5cpd at 20°[75, 76]. All of our subjects were able to detect the gratings when presented under focus and defocus conditions both centrally and in the peripheral retina.

In addition, the mfERG response of imposing +2/-2D defocus from central up to 20°in the periphery was investigated. We were concerned that peripheral regions should receive the same amount of imposed defocus as the central retina. Previous studies have demonstrated that emmetropic or hyperopic eyes showed myopic shifts whereas most myopes have hyperopic shifts in peripheral retina [75]. Yet, it is noticed that the refractive errors from central to peripheral 20°for low myopes (less than 2D) are relatively stable within the central 20°of the visual field [75, 77]. We are aware of the individual variation of peripheral refractive error beyond 20°of the visual field, but we assume the retina is experiencing roughly the same amount of defocus from ring 1 (0°) to ring 6 (19.2°) in our study.

The sign-dependent retinal activity was observed for the 0.24cpd stimulus in the peripheral regions but was not as obvious for the 4.8cpd stimulus. If the retina possesses a mechanism to differentiate the sign of defocus, there must be some clues for the retina to extract the sign of defocus. Our results suggest that the retina is more effective in extracting defocus under low SF than under high SF in peripheral regions rather than in the central region. Anatomically, the receptive field size increases with eccentricity, and the peripheral retina cannot resolve spatial details with very high SF. It is reasonable that the defocus decoding mechanism can respond to spatial stimuli with low SF rather than high SF. However, from our findings, we cannot be sure how the various defocus levels interact with the spatial details. That is, the defocus decoding mechanism may have preference for different SF under different optical defocus conditions. In terms of IC response to defocus, the trend was not as obvious as that of DC for the two SFs. A general trend of increased amplitude with +2D defocus and reduced amplitude with -2D defocus was observed for the 0.24cpd condition for rings 2 to 5.

Imposing defocus reduces the contrast of the retinal image and reduction in image contrast may potentially decrease the amplitude of the mfERG response. Previous study has shown imposing myopic defocus could reduce contrast sensitivity, especially at high SF [78]. In addition, the mfERG amplitude is decreased with a decrease of stimulus contrast without imposed optical defocus [41, 79]. Brown and Yap [79] showed that reductions of amplitudes are linear with little deviation in both foveal and peripheral regions. Yet our results showed some regions have increase in amplitudes after imposition of defocus. Both DC and IC amplitudes of MOFO mfERG were shown to be decreased with reducing contrast in previous studies [41, 47, 48]. Therefore, contrast reduction of the image after imposing defocus cannot fully account for the trend of mfERG amplitude response in low and high SF with +2/-2D defocus which should produce the same contrast reduction in terms of optical properties. Furthermore, the DC and IC amplitude responses of low SF across the retinal regions are comparable with the findings of *Ho et al*. [35]. Our data confirms their low SF data (regarding each hexagon as stimulus with very low SF) and extends their findings to high SF data, which in this study behaves differently to low SF stimuli.

### Does retinal image at different spatial frequency and retinal defocus affect myopia?

Results from animal studies have shown that emmetropization is spatial-frequency tuned [18–22] and myopia progression is related to exposure of hyperopic defocus [4, 36, 68, 72]. Both human and animal studies have shown that the eye can decode sign and magnitude of defocus [80], but it comes to the question how retina decodes the defocus. Our study indicates that the retina responses to defocus more vigorous in lower spatial frequency. Recent study suggested non-central retina is corresponding to eye growth [4, 68]. While the foveal region provides good spatial vision because of its high resolution, peripheral retina has lower resolution which would be insensitive to defocus in high spatial frequency as it cannot “resolve” the defocus. As the emmetropization is a local regulatory mechanism to manipulate the eye growth achieving a clear retinal image according to the visual signals, our findings provide evidence the human retina can differentiate sign of optical defocus at peripheral region, while it is more sensitive in lower spatial frequency in order to compensate the environmental influence. Further study is needed to confirm whether rate of eye growth is affected by spatial stimulus, given that the amount of defocus signal is kept the same.

## Conclusion

In this study, mfERG measures showed that outer and inner retina have different characteristics in processing spatial details. The peripheral retina can differentiate positive and negative defocus more effectively for low spatial frequencies than can the central retina. The human retina seems to have a decoding system for optical defocus, which is tuned for low spatial frequency, and is located in the retinal near periphery.

## Supporting Information

S1 Fig Global flash mfERG response for spatial frequency in high myope subjects. DC amplitude and IC amplitude of 12 age-matched subjects with spherical equivalent of -6.54±1.63D, was shown. The general trend of DC and IC amplitudes against spatial frequency for the high myope group were similar to those from low myope group.
(TIF)

S1 Dataset Raw data for experiment 1 and 2. (XLSX)
