# Supplementary figures and images for: Human Electroretinal Responses to Grating Patterns and Defocus Changes by Global Flash Multifocal Electroretinogram
Source: PLoS One. 2015 Apr 13;10(4):e0123480. doi: 10.1371/journal.pone.0123480 (PMC4395308; doi:10.1371/journal.pone.0123480)

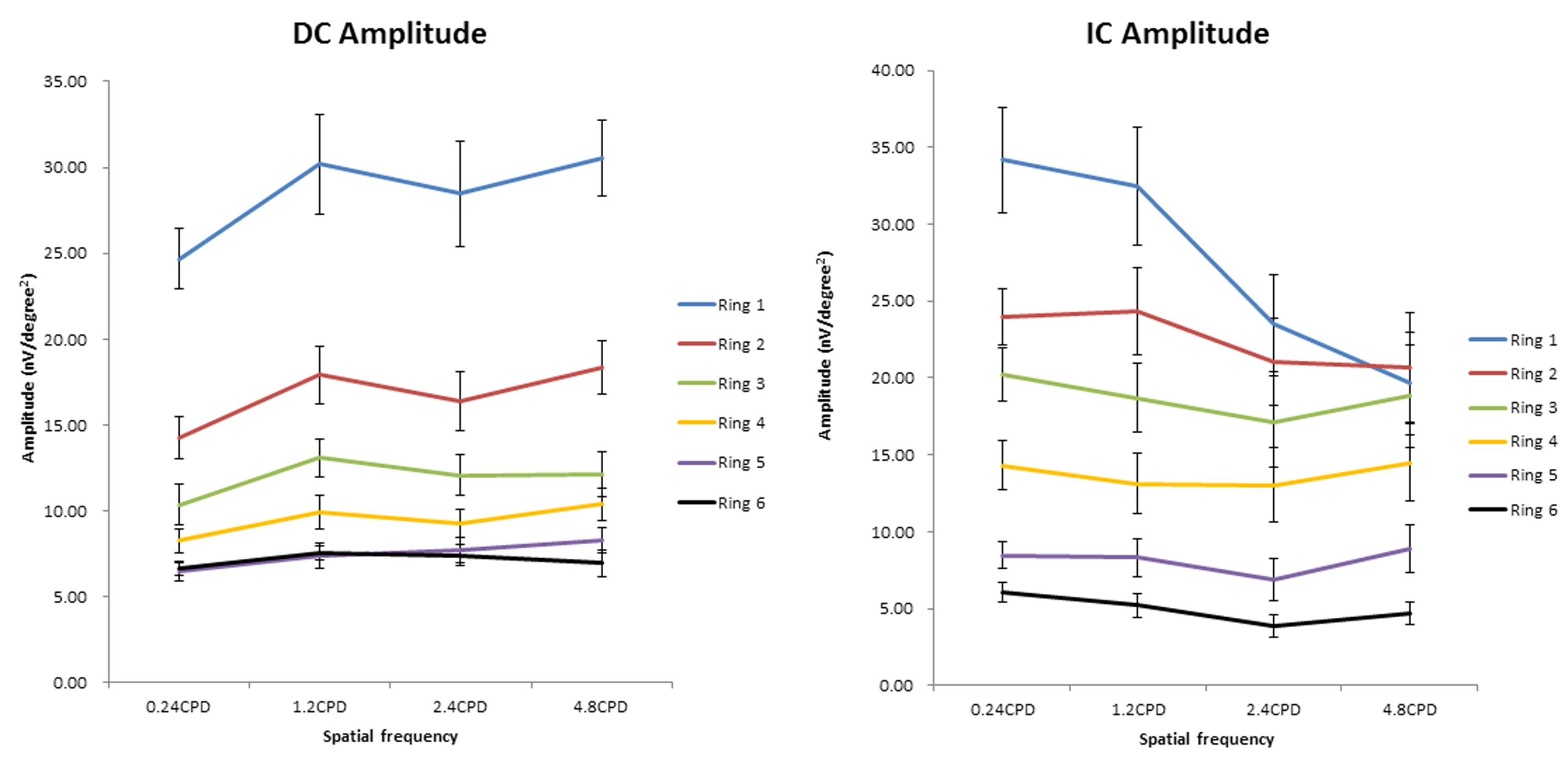

Supplement: S1 Fig — DC amplitude and IC amplitude of 12 age-matched subjects with spherical equivalent of -6.54±1.63D, was shown. The general trend of DC and IC amplitudes against spatial frequency for the high myope group were similar to those from low myope group. (TIF) [file pone.0123480.s001.tif]
